# Imaging the Micron: New Directions in Diagnosis with Ultra-High-Frequency Ultrasound

**DOI:** 10.3390/diagnostics14070735

**Published:** 2024-03-29

**Authors:** Rossana Izzetti, Marco Nisi

**Affiliations:** Department of Surgical, Medical and Molecular Pathology and Critical Care Medicine, University of Pisa, 56126 Pisa, Italy; marco.nisi@unipi.it

## 1. Introduction

In recent decades, advancements in medical imaging technologies have revolutionized diagnostic and therapeutic approaches, enhancing the precision and efficacy of healthcare interventions [1,2]. Among these cutting-edge technologies, ultra-high-frequency ultrasonography (UHFUS) has emerged as a powerful tool, offering unprecedented resolution and depth for imaging biological tissues [3]. This sophisticated ultrasound technique has gained an increasingly important role in several medical fields since its introduction into the clinical setting in the early 2000s [4,5,6,7]. Indeed, the technique has undergone significant changes due to the implementation of devices enabling the use of frequencies up to 100 MHz, which has led to a widespread application of UHFUS in all branches of medicine in which imaging of superficial structures at submillimeter resolution is required [8]. According to the current definition, UHFUS employs frequencies above 30 MHz, providing high-resolution imaging of anatomical structures at a microscopic level [9]. The applications of ultra-high-frequency ultrasonography in medicine are diverse and span various medical disciplines, including dermatology, rheumatology, pediatrics, and oral medicine. Recent studies have demonstrated how UHFUS can play a role in the diagnosis, management, surgical treatment, and follow-up of various pathologic conditions.

Hence, we provide a brief review on the applications of UHFUS and on the treatment opportunities related to this technique.

## 2. Uses of UHFUS Imaging by Medical Specialty

### 2.1. Dermatology

Dermatology was one of the first fields of application of ultrasonography; the technique was first employed in the late 1970s for the assessment of skin thickness [10,11,12] and has since then been used for the study of normal skin as well as several diseases and conditions with various etiologies. A recent systematic review by Lintzeri and coll. [13] summarized the evidence from the literature on epidermal thickness evaluated with different techniques, including UHFUS, and provided a stratification depending on the anatomic area investigated, gender, age, and ethnicity of the subjects, method of assessment, and skin phototype. Dini and coll. (Contributor 1) employed UHFUS to assess atopic dermatitis (AD) and its treatment outcomes. Importantly, in diagnosing AD, UHFUS can detect the pathognomonic sign of a subepidermal low-echogenic band (SLEB), which has been found to correlate with disease severity and treatment response. Moreover, SLEB measurement, along with the assessment of vascularity and epidermal thickness, might serve as an objective criterion to track responses to treatment. Additionally, detecting SLEB in non-lesional skin could indicate the presence of subclinical inflammation, potentially predicting the emergence of clinical lesions and emphasizing the need for proactive therapeutic intervention. Similarly, the assessment of pyoderma gangrenosum, a dermatosis of unknown origin, may benefit from UHFUS, as the technique may allow for the early identification of imaging biomarkers of lesion inflammatory status, as reported by Granieri and coll. (Contributor 2). Such biomarkers can support the detection, differential diagnosis, and treatment monitoring of PG and include epidermal and dermal morphology, vascularity, presence of edema, and aliasing phenomena.

UHFUS is also extremely valuable in the diagnostic algorithm of cutaneous autoimmune diseases [14]. Interestingly, the assessment and treatment monitoring of psoriasis represent a promising novel field of investigation [15]. Michelucci and coll. (Contributor 3) describe the use of UHFUS to evaluate psoriatic onychopathy severity.

When considering skin tumors, UHFUS may support the diagnosis and surgical treatment of several neoplastic conditions. Laverde-Saad and coll. [16] report that UHFUS provided accurate depth measurements of basal-cell carcinomas, especially if the lesions were above 1 mm. More specifically, the technique allowed for the assessment of tumor depth and margins, as well as for discriminating between aggressive and non-aggressive subtypes. Regarding melanoma skin lesions, a Cochrane systematic review by Dinnes and coll. [17] reported derived sensitivities of at least 83% and combined the assessment of three qualitative features, namely hypoechogenicity, homogeneity, and well-defined margins. In fact, UHFUS has been found to correlate the Breslow scale with ultrasonographic thickness [18,19]. The good correspondence between UHFUS and histology may improve the surgical treatment of these lesions, increasing the capability to obtain clear resection margins, which is of utmost importance in prognosis and in recurrence prevention. However, it should not be forgotten that UHFUS, combined with dermoscopy, can also prove beneficial in the diagnostic work-up of non-melanoma skin tumors [20,21,22]. 

Finally, additional evidence for using UHFUS to support clinical practice is provided by its role in esthetic medicine, where the technique is employed to monitor and assess treatment effects following anti-cellulite therapies, volumetric treatments, and discoloration correction [23,24]. Salvia and coll. (Contributor 4) utilized UHFUS to monitor hyaluronic acid filler distribution and nasolabial fold amelioration, potentially improving the outcomes of esthetic procedures. 

### 2.2. Rheumatology 

Most of the evidence on the applications of UHFUS in rheumatology revolves around the diagnosis and management of Sjögren’s syndrome [25]. The role of conventional ultrasonography of major salivary glands has been extensively investigated and was eventually recognized to improve the performance of the 2016 American College of Rheumatology/EUropean League Against Rheumatism (ACR/EULAR) classification criteria [26,27]. The application of UHFUS has permitted the evaluation of minor salivary glands, giving insights into a direct correlation between imaging and histology. Moreover, UHFUS-guided minor salivary gland biopsies have been proven to improve sampling accuracy and to reduce the risk of postoperative complications [28,29]. 

It is recognized that Sjögren’s syndrome incidence has two peaks, namely one after menarche (20–40 years of age) and one after menopause (50–60 years of age) [30]. However, some juvenile forms of Sjögren’s syndrome have been identified in patients below 18 years of age. Interestingly, in these cases, the presentation differs from that observed in adults, as xerostomia is significantly more common, while recurrent parotitis is often an early symptom [31,32,33]. Using diagnostic imaging for the evaluation of salivary glands in pediatric patients with suspected Sjögren’s syndrome may support the assessment of parenchymal and ductal damage, parenchymal inhomogeneity, and the presence of hypoechoic areas [34].

Indeed, the study by Marrani and coll. (Contributor 5) supports the usage of UHFUS in the monitoring of disease progression without exposing pediatric patients to surgical procedures. Another interesting aspect is the fact that UHFUS can be used to assess both the salivary and the lachrymal glands, which are both involved in the course of Sjögren’s syndrome [35,36,37]. Interestingly, symptoms of dry eye correlate poorly with clinical signs. While several techniques have been proposed for the assessment of lachrymal gland function, only recently has diagnostic imaging been introduced to evaluate glandular size and inflammation [38].

According to Fulvio and coll. (Contributor 6), assessing the lachrymal glands by means of UHFUS may improve the phenotyping of affected patients, providing a more comprehensive understanding of overall glandular involvement.

A pivotal role of UHFUS has also been proposed in the measurement of epidermal thickness and echogenicity in evaluating patients with systemic sclerosis as a complementary method to standard assessment, as reported by Di Battista and coll. (Contributor 7).

### 2.3. Pediatrics

UHFUS finds several applications in pediatric dermatological medicine due to the lack of ionizing radiation, which is extremely important when managing young patients [39]. As reported by the pictorial review by Ait Ichou et al. [40], the technique can be utilized for the assessment of cutaneous lesions, musculoskeletal imaging, and vascular malformations. UHFUS has been reported to help in the evaluation of soft tissues and superficial structures, and it can potentially be employed intraoperatively to evaluate tumor margins and small structures.

It appears noteworthy that abdominal imaging may also benefit from UHFUS application. In fact, previous evidence supports the use of UHFUS for the study of the bowel wall in the course of Hirschsprung disease, a congenital disorder which compromises distal bowel innervation [41]. In these patients, nerve fiber hypertrophy as well as absent or impaired ganglia are observed as a result of an anomaly in neural crest cell migration during fetal development [42]. While the gold standard for diagnosis is rectal suction biopsy, it has been hypothesized that ultrasonography may help to rule out the presence of the disease [22]. Erlöv et al. (Contributor 8) employed UHFUS to study the bowel wall in Hirschsprung’s disease to assess the thickness of bowel wall layers. In the study by Hawez and coll. (Contributor 9), UHFUS was able to discriminate between bowel wall muscular layers in children with Hirschsprung’s disease, with a good correlation with histology. Previous research focused on the ability of UHFUS to discriminate between aganglionic and ganglionic bowel wall [36]. However, the study by Evertsson and coll. (Contributor 10) highlights that as of now, a dedicated UHFUS probe for rectal ultrasound is not available, thus highlighting the unmet need for a specific device for bowel assessment in pediatric patients.

Functional imaging, aimed at detecting the physiology and dynamics of anatomical structures, is gaining increasing importance. Interestingly, Alan and coll. (Contributor 11) applied UHFUS to study the dynamics of breastfeeding in neonates to detect variations in tongue positioning and movement in subjects with ankyloglossia versus healthy ones. In this field, UHFUS can prove beneficial in evaluating the severity of ankyloglossia and thus provide indication for frenotomy by specifically assessing the amplitude of anterior tongue movement and sucking efficacy during breastfeeding.

From this perspective, the ability to study the dynamics of the digestive system represents an advance in terms of diagnostic performance. Moreover, UHFUS may be helpful not only in assessing the presence of disease or impaired function, but also in providing guidance for surgical procedures. As reported by Guo et al. [43], the performance of UHFUS-guided endoscopic retrograde appendicitis therapy may improve treatment outcomes by allowing for real-time monitoring during stent placement. 

The evidence in the field of pediatrics thus supports a growing application of UHFUS both for diagnostic and surgical purposes due to it having the major advantage of avoiding radiation exposure to pediatric patients.

### 2.4. Oral Medicine

Research on intraoral ultrasound has seen an increasing interest since the late 1990s, when the technique started to be employed for the assessment of tongue cancer [44,45,46,47]. Importantly, the parameter of depth of invasion was established as a predictor of the presence of lymph node metastases [48,49,50] and was added as a T-category modifier to the 8th edition of the American Joint Committee on Cancer criteria in 2017 [51,52,53]. Several studies have investigated the ability of diagnostic imaging to evaluate tumor dimensions [54,55,56]; a recent systematic review compared the diagnostic accuracy of magnetic resonance, computed tomography, and ultrasonography in assessing tumor dimensions preoperatively [57]. For magnetic resonance, the sensitivity was >80% and the specificity was >75% in all the studies included in the review, while computed tomography was reported in one study to have a sensitivity of 68.75% and a specificity of 77.78%. Ultrasonography showed the highest mean values for both sensitivity (>91%) and specificity (100%). It is noteworthy that ultrasonography proves extremely valuable in the case of small lesions (<4 mm) that may not be detectable with other techniques [58].

Although different ultrasound frequencies and protocols have been employed in the literature, the systematic review by Nisi and coll. (Contributor 12) confirms that ultrasonography to assess the depth of invasion is a sensitive tool with good correlation with histology. Indeed, UHFUS has the unique advantage of providing a higher resolution compared to conventional ultrasonographic techniques, thus improving diagnostic accuracy [59]. Indeed, its reduced invasiveness, repeatability, and the absence of exposure to ionizing radiation make the technique widely applicable to oral lesions. While research on oral cancer appears extremely robust, it should be highlighted that the versatility of UHFUS also allows for the investigation of other oral diseases and conditions, thus supporting the diagnosis, treatment, and follow-up of several oral lesions [60].

### 2.5. Other Applications

The UHFUS technique has been reported to effectively provide imaging of peripheral nerves [61,62]. Apart from structural analysis, UHFUS can be employed to characterize nerve echogenicity, which can help to discriminate between normal and abnormal nerves by applying the nerve density index, as reported by Jerman and coll. (Contributor 13).

UHFUS can also support the monitoring of lipohypertrophy onset in patients with diabetes treated with insulin injections. According to Yu and coll. (Contributor 14), these lesions can be detected earlier when using UHFUS, allowing practitioners to correct the injection method and to avoid injection at the lesion site.

The application of high-intensity focused ultrasound for surgical purposes is described by Liu and coll. (Contributor 15), with a focus on the ablation of juvenile cystic adenomyosis lesions. Ablation is the technique of choice as surgery may damage the muscular tissues surrounding the cystic adenomyosis lesions, potentially contributing to an increase in the risk of uterine rupture during pregnancy and causing iatrogenic endometriosis. Treatment with high-intensity focused ultrasound allows for the safe treatment of cystic adenomyosis, decreasing lesion dimensions and vascularization while also resolving lesion-associated dysmenorrhea.

## 3. Conclusions

UHFUS holds promise for several medical fields due to its advantages of providing real-time imaging, avoiding exposure to ionizing radiation, and offering repeatability in the follow-up examination of lesions. As highlighted by the contributions to this Special Issue, the current research supports the increasing use of this technique in the diagnosis, prognosis, treatment, and follow-up of several diseases and conditions.
